# *In vivo* neuron-specific expression of *C. elegans* reprogramming factor orthologs does not alleviate age-related cognitive decline

**DOI:** 10.17912/micropub.biology.001304

**Published:** 2024-08-29

**Authors:** Erik Toraason, Rachel Kaletsky, Coleen Murphy

**Affiliations:** 1 Lewis-Sigler Institute for Integrative Genomics, Princeton University; 2 Department of Molecular Biology, Princeton University

## Abstract

Overexpression of the OSK(M) (Oct4, Sox2, Klf4, with or without cMyc) pluripotency factors have shown promise in rejuvenating the function of aged neurons. To test whether this intervention could also ameliorate age-associated cognitive decline, we used a doxycycline inducible system to overexpress the *C. elegans* OSK orthologs specifically in aging *C. elegans* neurons. We find that OSK does not improve short-term associative memory or extend lifespan and can further disrupt chemotaxis behavior. Taken together, our data suggest that OSK-mediated partial reprogramming may have deleterious effects on post-mitotic neurons that function in cognitive processes.

**Figure 1. Effect of neuronal OSK on C. elegans lifespan and cognitive aging:**
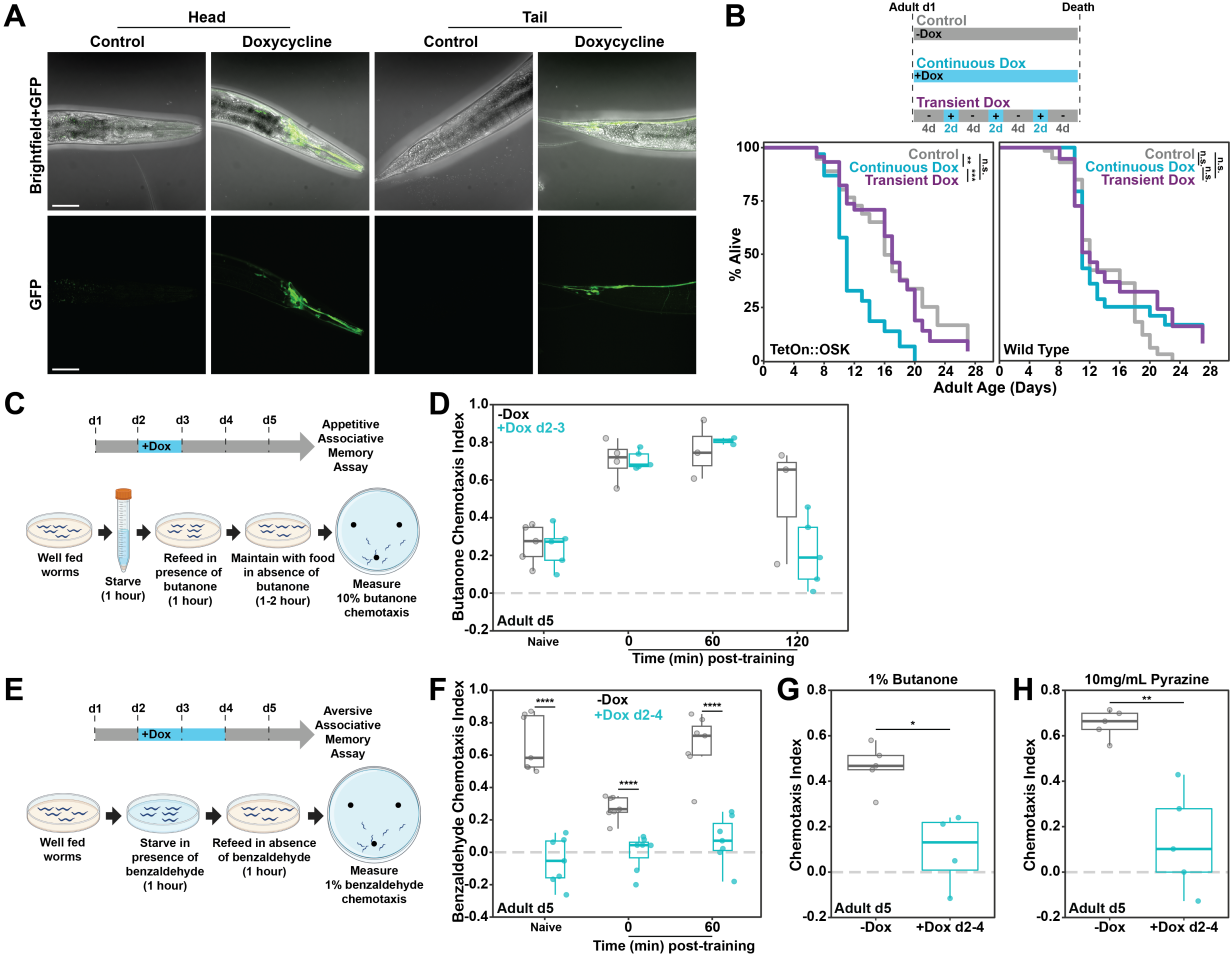
A) A doxycycline-inducible genetic construct enables neuronal expression of *C. elegans* OSK factor orthologs and GFP with temporal control. Images displayed are maximum intensity projections of representative head and tail neurons following exposure to control buffer or doxycycline (see Methods). Scale bars indicate 50μm. B) Lifespan assay (1 replicate) of CQ751 worms and wild-type non-array carrying siblings never exposed to doxycycline (control), exposed to doxycycline throughout adult life (continuous dox), or transiently exposed to doxycycline for 48h every 4 days (transient dox). P values were calculated by Log-Rank test with Bonferroni-Holm correction for multiple comparisons. C) Diagram of appetitive memory assay and timing at which CQ819 worms were exposed to doxycycline (24 hours beginning at adult day 2). D) Appetitive short-term memory assay of day 5 adult CQ819 worms exposed to doxycycline for 24 hours beginning at adult day 2. P values were calculated by ANOVA with Tukey's HSD tests. 1 representative replicate of n=2 independent experiments is displayed. E) Diagram of aversive memory assay and timing at which CQ819 worms were exposed to doxycycline (48 hours beginning at adult day 2). F) Aversive short-term memory assay (1 replicate) of day 5 adult CQ819 worms exposed to doxycycline for 48 hours beginning at adult day 2. P values were calculated by ANOVA with Tukey's HSD tests. G) 1% butanone chemotaxis assay (1 replicate) of day 5 adult CQ819 worms exposed to doxycycline for 48 hours beginning at adult day 2. P values were calculated by Student's t-test. H) 10mg/mL pyrazine chemotaxis assay (1 replicate) of day 5 adult CQ819 worms exposed to doxycycline for 48 hours beginning at adult day 2. P values were calculated by Student's t-test. In all panels, n.s. = p>0.05, * = p<0.05, ** =p<0.01, ***=p<0.001, **** = p<0.0001.

## Description

Aging is defined by the progressive loss of cell, tissue, and organ function over time, culminating in death. One of the most debilitating aspects of aging in humans is cognitive decline, as memory function is vital for autonomy and individual identity. Recent work in rodent models has demonstrated that transient overexpression of the Yamanaka pluripotency factors OSK(M) (Oct4, Sox2, Klf4, with or without cMyc) (Ocampo et al., 2016; Takahashi & Yamanaka, 2006) rejuvenates the function of aged peripheral neurons (Lu et al., 2020) , increases adult neurogenesis (Xu et al., 2024) , and may improve some memory functions of the hippocampus (Horvath et al., 2023) . However, overexpression of OSK(M) also carries risk, as this intervention can produce teratomas and cause hepatic and intestinal failure *in vivo* (Abad et al., 2013; Parras et al., 2022) . Thus, identifying the downstream effectors that mediate OSK(M) neuron rejuvenation may enable the development of targeted therapies to treat age-associated cognitive decline while mitigating these risks.

The nematode *Caenorhabditis elegans* is a powerful system in which to dissect the fundamental molecular mechanisms of cognitive aging (Kauffman et al., 2010; Lakhina et al., 2015; Stevenson et al., 2023) . *C. elegans* exhibits age-associated cognitive decline relatively early in life; by the fifth day of adulthood, they lose the ability to form long-term memories, and short-term memory is significantly decreased (Kauffman et al., 2010) . To assess whether overexpression of reprogramming factors is sufficient to ameliorate age-associated cognitive decline in *C. elegans* , we created a strain expressing the *C. elegans* orthologs of the OSK factors (Oct4 = *ceh-6* , Sox2 = *sox-2* , Klf4 = *klf-3* ) (Hsieh et al., 2017; Kagias et al., 2012) under the control of a neuron-specific doxycycline inducible promoter (Mao et al., 2018) ( Figure 1A ). Some larvae expressing the OSK transgenes exhibited developmental defects when raised in the presence of doxycycline, therefore we limited our future experiments to adult-only induction of OSK.

Neuronal cues regulate lifespan in *C. elegans* (Apfeld & Kenyon, 1999; Alcedo & Kenyon, 2004; De-Souza et al., 2023) . To assess whether overexpression of OSK in neurons affected lifespan, we induced neuron-specific OSK expression throughout adult lifespan (“continuous”) or transiently in discrete periods ( Figure 1B ). We found that transient induction of OSK did not significantly affect lifespan ( Figure 1B ), but continuous induction of OSK orthologs reduced adult lifespan ( Figure 1B ). This phenotype was not due to doxycycline toxicity, as wild type worms exhibited normal lifespan in the presence of doxycycline ( Figure 1B ). Our data parallels recent work from the Ocampo group, which found that expression of *C. elegans* reprogramming factors in the adult soma failed to extend or reduced *C. elegans* lifespan and further exerted deleterious effects on reproduction and larval development (Kamaludeen et al., 2024) .

To test whether transient OSK expression could delay cognitive aging, we induced expression of OSK beginning at the second day of adulthood, when worms first exhibit phenotypes of cognitive decline (Kauffman et al., 2010) . We then tested the worms' ability to learn and form short-term associative memories on the fifth day of adulthood in appetitive ( Figure 1C ) and aversive ( Figure 1E ) assays. In the appetitive short-term memory assay, in which worms are trained to associate the olfactant butanone with the presence of food ( Figure 1C ), we observed no significant effect of OSK induction on either learning or short-term memory relative to uninduced controls ( Figure 1D ).

We next asked whether transient OSK might influence the formation and/or retention of aversive memories using an assay in which worms are trained to associate the olfactory attractant benzaldehyde with starvation ( Figure 1E ) (Lin et al., 2010; Nuttley et al., 2002) . We found that naive chemotaxis to benzaldehyde was reduced following OSK induction ( Figure 1F ). Further, OSK-expressing worms failed to form an association between benzaldehyde and starvation, as indicated by the reduced chemotaxis index after training in the uninduced control condition ( Figure 1F ). These results suggest that the worms' ability to sense benzaldehyde may be disrupted following OSK induction, precluding the formation of an aversive associative memory. To test whether detection of other olfactants was also impeded by OSK expression, we assessed the effect of OSK on chemotaxis to low concentration butanone ( Figure 1H ) and pyrazine ( Figure 1G ), which worms are naively attracted to. We found that attraction to both pyrazine and butanone was reduced following neuronal OSK expression ( Figure 1G, H ). Both benzaldehyde and butanone are sensed by the AWC olfactory neurons (Bargmann et al., 1993) , while pyrazine is sensed by the AWA olfactory neuron (Bargmann et al., 1993) . Our data therefore demonstrate that the function of multiple olfactory neurons is disrupted following prolonged (48 hours) induction of *C. elegans* reprogramming factors.

In summary, our results suggest that neuronal OSK expression does not meaningfully ameliorate cognitive aging or extend lifespan in *C. elegans* and can further disrupt chemosensation. As the *C. elegans* soma is entirely post-mitotic, the lack of apparent rejuvenation by OSK in *C. elegans* both observed in our study and by Kamaludeen et al. suggest that terminally-differentiated cells may not benefit from this intervention. It is possible that pluripotency is regulated differently in *C. elegans* than in other species; we do not favor this hypothesis, however, as the OSK(M) pluripotency factors are widely conserved from mammals to invertebrates (Rosselló et al., 2013) and the worm OSK orthologs are required for a cellular transdifferentiation event that occurs during larval development (Kagias et al., 2012) . Our results contrast with the dramatic regeneration and rejuvenation of injured or aged mouse post-mitotic retinal ganglion cells (RGCs) by OSK (Lu et al., 2020) . This rejuvenation is dependent upon DNA demethylation (Lu et al., 2020) , and depletion of the DNA cytosine methyltransferase DNMT3a is sufficient to induce RGC regeneration (Tai et al., 2023) . As *C. elegans* lacks cytosine methylation (Simpson et al., 1986) , the absence of apparent benefit of this intervention in worms raises the hypothesis that the benefits of OSK may be specific to organisms and cell types in which changes in DNA methylation contribute to the aging process. Taken together, our work suggests that future research on the efficacy of OSK(M) in cognitive aging may be best targeted at neurogenic processes, rather than in restoring healthy functions to aging neurons.

## Methods

*C. elegans* Strains and Culture Conditions

Strains were maintained under standard conditions at 20°C high growth medium (HG) agar plates [3 g/L NaCl, 20 g/L Bacto-peptone, 30 g/L Bacto-agar, 4 mL/L cholesterol (5 mg/mL in ethanol), 1 mL/L 1M CaCl _2_ , 1 mL/L 1M MgSO _4_ , and 25 mL/L 1M KPO _4_ (pH 6.0)] seeded with OP50 *E. coli* bacteria. Populations of worms were synchronized by bleaching gravid adults [alkaline-bleach solution: 2.5mL 1M KOH, 6mL hypochlorite bleach, 41.5mL ddH2O] followed by two washes with M9 buffer [6 g/L Na _2_ HPO _4_ , 3 g/L KH _2_ PO4, 5 g/L NaCl, 1 mL/L 1M MgSO4 in ddH2O]. For appetitive and aversive learning assays using aged worms, worms were transferred to HG plates supplemented with 51μM FuDR at L4 stage. Worms on HG+FuDR plates were transferred to HG plates 24 hours before assays were performed. Doxycycline treatment was administered by placing 400mL of 1ng/μL doxycycline hyclate in M9 buffer onto 60mm plates seeded with OP50 or 556mL of 2ng/μL doxycycline hyclate in M9 buffer onto 10cm plates seeded with OP50 . In all cases, NGM and HG plates were completely dry when worms were transferred to them.

Cloning and generation of transgenics

Plasmids to express *ceh-6* (pET020), *klf-3* (pET022), and *sox-2* (pET024) under the control of the doxycycline inducible TRE::Δ *pes-10p* promoter were constructed by amplifying *C. elegans* genomic DNA of each locus to replace the GFP sequence in pTC358 (TRE::Δ *pes-10p* ::GFP) by Gibson assembly (New England Biolabs). *ceh-6* was amplified using primers (forward 5'-ATGCTCATACCTTCGTCGTCA-3', reverse 5'- CTATTGTTGTCTCGGGCTCTG-3'), *klf-3 a* was amplified using primers (forward 5'- ATGACATCGCCAAACATTTTT-3', reverse 5'- CTAGATTGTGCTATGGCGCTT-3'), and *sox-2 b* is amplified using primers (forward 5'-ATGCACAATTCTGAAATCAGC-3', reverse 5'- TTAAGAGGTAACATGGGATTG-3'). Neuron-specific TetOn effector plasmid pET026 ( *rgef-1p* ::rtetR-QFAD) was cloned by amplifying the *rgef-1* promoter (forward 5'- cgagtcaactgaaatccgttc-3', reverse 5'-cgtcgtcgtcgtcgatgccgt-3') to replace the *rpl-28p* sequence in pTC374 ( *rpl-28p* ::rtetR::QFAD) using Gibson Assembly (New England Biolabs). The sequences of all plasmids were confirmed by sequencing (Plasmidsaurus). CQ751 carrying a doxycycline-inducible neuron-specific OSK extrachromosomal array was generated by injecting N2 hermaphrodites with pET020 (7.9 ng/μL), pET021 (6.6 ng/μL), pET024 (4.7 ng/μL), pET026 (5 ng/μL), pTC358 (5 ng/μL), and pCFJ90 (2 ng/μL). CQ751 was maintained by picking mCherry+ worms. During maintenance, the array spontaneously integrated into the genome, as observed by inheritance of the transgenes in 100% of progeny. This integrated strain was named CQ819 .

Short-term appetitive associative memory assay

Appetitive associative memory assays were performed as described in Kauffman et al. PLoS Bio. 2010. In brief, worms raised on HG plates were washed 3x with M9 buffer to remove any food and then were starved for 1 hour in 3mL of M9 buffer. The worms were then transferred to 10cm NGM plates seeded with OP50 and streaked on the lid with 18μL of 10% 2-butanone in ethanol for 1 hour. Following butanone training, the worms were maintained on 10cm NGM plates in the absence of butanone. Standard chemotaxis assays (Bargmann et al., 1993) on 10cm NGM plates measuring *C. elegans '* preference for 10% 2-butanone in ethanol as compared to 100% ethanol (as measured by first choice, as worms are paralyzed at each spot with sodium azide) were performed on untrained populations (“naïve”), immediately after butanone training (0 min), and 60 or 120 minutes after butanone training. The chemotaxis index was calculated as (# worms at butanone - # worms at ethanol)/(total worms - worms at origin).

Short-term aversive associative memory assay

Aversive associative memory assays were adapted from established methods (Lin et al., 2010; Nuttley et al., 2002) . In brief, worms raised on HG plates were washed 3x with M9 buffer and then were placed on NGM plates with no bacterial food with 2μL of 100% benzaldehyde spotted on the lid. The worms were then transferred to 10cm NGM plates seeded with OP50 in the absence of benzaldehyde. Standard chemotaxis assays (Bargmann et al., 1993) on 10cm NGM plates measuring *C. elegans* preference for 1% benzaldehyde in ethanol as compared to 100% ethanol (as measured by first choice, as worms are paralyzed at each spot with sodium azide) were performed on naive populations, immediately after benzaldehyde starvation training (0 min), and 60 minutes after butanone training. The chemotaxis index was calculated as (# worms at benzaldehyde - # worms at ethanol)/(total worms - worms at origin).

Butanone and pyrazine chemotaxis

Standard chemotaxis assays (Bargmann et al., 1993) on 10cm NGM plates measuring *C. elegans* preference for 1% butanone or 10mg/mL pyrazine in ethanol as compared to 100% ethanol (as measured by first choice, as worms are paralyzed at each spot with sodium azide) were performed on naive populations. The chemotaxis index was calculated as (# worms at butanone or pyrazine - # worms at ethanol)/(total worms - worms at origin).

Lifespan assay

Bleach-synchronized CQ751 eggs were placed to NGM plates seeded with OP50 . Beginning at adult day 1, worms were placed onto 60cm NGM plates seeded with OP50 and spotted with 400μL M9 buffer or 400μL 1ng/μL doxycycline in M9 buffer. All plates were allowed to completely dry before worms were placed on them. ‘Control' worms were maintained exclusively on plates with M9 alone, while ‘continuous doxycycline' treated worms were maintained in the presence of doxycycline. ‘Transient doxycycline' treated worms were transferred to doxycycline plates for 48 hours beginning at the adult day 4, adult day 10, adult day 16, and so on until all worms were dead.

Microscopy

Worms were synchronized by bleaching onto NGM plates seeded with OP50 . Day 1 adults were transferred to 10cm NGM plates seeded with OP50 and treated with 556μL of M9 (control) or 2ng/μL doxycycline hyclate in M9 buffer. 24 hours later, worms were washed from these plates with M9 buffer and were plated on 1% agarose pads in M9 buffer and were paralyzed with 1% sodium azide. Images were acquired using a Nikon AXR confocal microscope at 60x magnification with 1μm z-stacks. Images were processed using NIS-Elements and FIJI.

Statistics

All statistical analyses were performed using R (v4.3.2). Data wrangling was performed using the tidyverse (v2.0.0), reshape2 (v1.4.4), and DescTools packages (0.99.53). Lifespan survival analysis was performed using the survival (v3.5-7) and ggsurvfit (v1.0.0) packages. Specific statistical tests used are denoted in the figure legend.

## Reagents

**Table:** 

Strain	Genotype	Available from
CQ751	wqEx85 [Pmyo-2::mCherry + Prgef-1::tetR-QFAD::P2A::mKate::T2A::Tetr-pie-1 + TRE::ΔPpes-10::sox-2a + TRE::ΔPpes-10::klf-3 + TRE::ΔPpes-10::ceh-6 + TRE::ΔPpes-10::GFP] )	This study
CQ819	wqIs8 [Pmyo-2::mCherry + Prgef-1::tetR-QFAD::P2A::mKate::T2A::Tetr-pie-1 + TRE::ΔPpes-10::sox-2a + TRE::ΔPpes-10::klf-3 + TRE::ΔPpes-10::ceh-6 + TRE::ΔPpes-10::GFP] )	This study
N2	Wild type	*Caenorhabditis* Genetics Center (CGC)

**Table:** 

Plasmid	Description	Available from
pET021	TRE-delta-pes-10-ceh-6	This study
pET022	TRE-delta-pes-10-klf-3a	This study
pET024	TRE-delta-pes-10-sox-2b	This study
pET026	Prpl-28::rtetR-QFAD::P2A::mKate::T2A::tetR-pie1	This study
pCFJ90	Pmyo-2::mCherry	Addgene
pTC358	TRE-delta-pes-10-GFP	Addgene
pTC374	Prpl-28::rtetR-QFAD::P2A::mKate::T2A::tetR-pie1	Addgene
